# Increased KLRG1 and PD-1 expression on CD8 T lymphocytes in TB-IRIS

**DOI:** 10.1371/journal.pone.0215991

**Published:** 2019-04-25

**Authors:** Odin Goovaerts, Marguerite Massinga-Loembé, Pascale Ondoa, Ann Ceulemans, William Worodria, Harriet Mayanja-Kizza, Robert Colebunders, Luc Kestens, for the TB-IRIS Study Group

**Affiliations:** 1 Department of Biomedical Sciences, Institute of Tropical Medicine, Antwerp, Belgium; 2 Centre de Recherches Médicales de Lambaréné (CERMEL), Albert Schweitzer Hospital, Lambarene, Gabon; 3 Institut für Tropenmedizin, Universität Tübingen, Tübingen, Germany; 4 Department of Global Health and Amsterdam Institute for Global Health and Development, Academic Medical Centre, Amsterdam, The Netherlands; 5 Department of Medicine, Mulago Hospital, Kampala, Uganda; 6 Infectious Diseases Institute, Makerere University College of Health Sciences, Kampala, Uganda; 7 Infectious Diseases Network for Treatment and Research in Africa (INTERACT), Kampala, Uganda; 8 Department of Clinical Sciences, Institute of Tropical Medicine, Antwerp, Belgium; Central University of Tamil Nadu, INDIA

## Abstract

**Background:**

Tuberculosis-associated immune reconstitution inflammatory syndrome (TB-IRIS) is an inflammatory complication in HIV-TB co-infected patients receiving antiretroviral therapy (ART). The exact contribution of T cells, natural killer (NK) cells, and monocytes to TB-IRIS development remains unclear. Here, we studied the expression of exhaustion markers on lymphocytes at different intervals during ART.

**Methods:**

We compared 13 HIV-TB patients who developed TB-IRIS with 13 patients who did not (HIV^+^TB^+^), 13 HIV-patients without TB (HIV^+^TB^-^) and 9 HIV/TB-negative controls (HIV^-^TB^-^). Patients did not differ in age, gender, or CD4-count prior to ART. Frozen peripheral blood mononuclear cells, collected before ART and during 3 months and 9 months of ART, were analysed using flow cytometry. We examined expression of KLRG1, PD-1 and IL-27R on CD4^+^ and CD8^hi^ T cells, as well as CD3-negative CD8^lo^ lymphocytes as an approximate subset of NK cells. In addition, expression of TLR2, TLR4, IL1RL1, and TRAILR on CD14^+^ monocytes were investigated.

**Results:**

Prior to ART, TB-IRIS patients had higher percentages of CD8^hi^ T cells that are KLRG1^+^PD-1^+^ compared to each control group (p≤0.034). Though PD-1 expression decreased during ART in all groups (p≤0.026), the percentage KLRG1^+^PD-1^+^CD8^hi^ T cells remained higher in TB-IRIS patients after 3 months of ART (p≤0.013). Though these patterns were less pronounced in CD3^-^CD8^lo^ lymphocytes, the percentage of KLRG1^+^ cells was higher in TB-IRIS patients prior to ART (p≤0.043). In contrast, no clear differences could be observed for CD4^+^ T cells or monocytes.

**Conclusion:**

TB-IRIS is preceded by a high level of exhausted (KLRG1^+^PD-1^+^) CD8^hi^ T cells, which persists during 3 months of ART. This trait is potentially mirrored in a subpopulation of NK cells, but not CD4^+^ T cells. Since a dysfunctional CD8^+^ lymphocyte compartment could predispose patients to TB-IRIS, the functional role of these cells prior to TB-IRIS development should be further explored.

## Introduction

During successful antiretroviral therapy (ART), a subgroup of HIV patients with a tuberculosis (TB) co-infection are at risk of developing a complication called paradoxical TB-associated immune reconstitution inflammatory syndrome (TB-IRIS) [1]. TB-IRIS is characterized by worsening symptoms of TB, despite an effective initial response to concurrent TB-treatment [2]. Marked by tissue-destructive inflammation and a wide array of symptoms, patients often require additional therapy which increases the cost of patient care [3]. Moreover, diagnosis of TB-IRIS still mainly relies on clinical examinations and is often difficult to distinguish from other complications. Thus, there is an urgent need for reliable laboratory markers to predict this syndrome, since the immune-pathogenesis of TB-IRIS is still not well understood [4].

TB-IRIS typically develops within the first 3 months after starting ART, with the majority of cases occurring before 1 month when CD4^+^ T cells are being replenished [5,6]. Known risk factors of TB-IRIS include a high TB-antigen burden, a short interval between TB treatment and ART and, most importantly, a low CD4^+^ T cell count prior to ART initiation [7–9]. It should be noted, however, that not all HIV-TB patients under similar conditions of immunosuppression develop TB-IRIS. One major characteristic of TB-IRIS is the occurrence of a cytokine storm during the peak of inflammation [10–13]. Thus, the idea that IRIS involves an atypical restoration of immune responses to TB has gained acceptance [5,14,15]. Whereas a dominant role of innate immune cells in the inflammatory cascade during TB-IRIS has become increasingly apparent [11,12,16,17], it still remains unclear which innate or adaptive factors prime the immune system to over-react before ART is administered.

A number of previous TB-IRIS studies have reported pre-ART anomalies in cells belonging to either the innate or the adaptive arm of the immune system. On one hand, increased frequencies of activated CD14^+^ monocytes have previously been reported as a predictor of TB-IRIS [18]. In addition, TB-IRIS patients have been reported to have higher toll-like receptor (TLR)-2 expression on monocytes [19], and a higher degranulation capacity of natural killer (NK) cells prior to starting ART [20]. On the other hand, TB-IRIS patients have been described to have higher pre-ART percentages of activated CD4^+^ T cells and CD8^+^ T cells [21,22]. Nonetheless, other studies did not observe such T cell activation [23,24], whereas we previously observed lower levels of HLA^+^CD38^+^ CD8^+^ T cells in TB-IRIS patients prior to ART [25].

As the predisposing role of these cells in TB-IRIS thus remains elusive, more research is required to fully understand the phenotypic characteristics of T cells, NK cells and monocytes before ART is administered. One of the hallmarks of long term HIV and TB infection is T cell exhaustion and senescence, linked to higher expression of PD-1, KLRG1 and interleukin-27 receptor (IL-27R) on CD4^+^ T cells, CD8^+^ T cells and NK cells [26–28]. Exhausted cells are known to be less functional and could allow the accumulation of antigenic stimuli prior to ART, thus priming the innate immune system for subsequent TB-IRIS development. We therefore hypothesised that higher levels of T cell exhaustion prior to ART would predispose HIV-TB patients with severe immunosuppression to develop TB-IRIS. In addition, we hypothesized that this could be reflected in the expression of TLR related proteins on monocytes [29]. Nested within a large prospective cohort of TB-IRIS patients, this case-control study describes higher pre-ART percentages of CD8^hi^ T cells and CD3^-^CD8^lo^ lymphocytes with an exhausted phenotype in TB-IRIS patients, which persist during 3 months of ART. In contrast, no differences were observed for CD4^+^ T cells, nor the expression of TLR-related proteins on monocytes.

## Methods

### Study population

The clinical spectrum of HIV-TB and TB-IRIS was studied in a prospective observational study at Mulago Hospital, Kampala, Uganda between 2007 and 2012 [7, 30]. The study enrolled HIV-TB co-infected adults (HIV^+^TB^+^) who were being treated for active TB infection for less than 2 months, as well as HIV-patients without clinical signs of TB co-infection (HIV^+^TB^-^). In addition, a group of HIV-TB negative controls (HIV^-^TB^-^) was recruited. Exclusion criteria included: pregnancy, prior use of ART and Grade 3 renal or liver abnormalities. All HIV-patients were started on a non-nucleoside reverse transcriptase inhibitor-based ART according to Ugandan national guidelines. Including adherence preparation for HIV^+^TB^+^ patients, the median interval between starting TB-treatment and ART was 6 weeks. HIV-patients periodically had blood samples taken for a period of 10 months to monitor paradoxical TB-IRIS development. Sixty (24%) out of 254 HIV-TB co-infected patients developed IRIS (TB-IRIS patients), whereas HIV^+^TB^+^ patients who did not develop IRIS-related symptoms served as non-IRIS controls. For the purpose of the current study, a subset of patients were randomly selected within each patient group, based on the availability of cryopreserved PBMC samples. Samples taken before initiation of ART, at 3 months and at 9 months after starting ART were analysed, whereas HIV-negative controls had samples taken only once.

### Definitions

*Mycobacterium tuberculosis* infection was diagnosed according to the TB/HIV WHO guidelines [31]. Investigations to confirm TB infection included: clinical examination, sputum smear microscopy for acid-fast bacilli, abdominal ultrasounds, chest X-rays, and mycobacterial culture of sputum, aspirate or effusion if available. TB-IRIS cases were classified by a committee of two co-authors (RC and WW) after reviewing all suspected TB-IRIS cases evaluated by the study physicians according to the International Network for the Study of HIV-associated IRIS (INSHI) clinical case-definition [1]. TB-IRIS was diagnosed when patients presented with at least 1 major criterion (e.g. enlarged lymph nodes) or 2 minor criteria (e.g. fever and cough) and other explanations such as treatment failure were excluded.

### Flow cytometry

Peripheral blood mononuclear cells (PBMCs) were collected from all patients and cryopreserved in liquid nitrogen. PBMCs were consequently thawed, counted and checked for viability with trypan-blue, incubated with Human BD Fc Block™ (Becton Dickinson (BD)), stained with fluorescently labelled antibodies and fixed with 1% paraformaldehyde in PBS before measuring with a BD FACSVerse™ eight-colour flow cytometer (BD). Two antibody panels were used to determine the phenotype of either lymphocytes or monocytes, respectively; lymphocyte panel: CD3-APC-H7, CD4-PerCP-Cy5.5, CD8-BV510, PD-1-BV421 (BD), CD45RO-FITC (Miltenyi), KLRG1-APC (ebioscience), and IL27R-PE (R&D systems); Monocyte panel: CD14-Viogreen, TLR2-PE-Vio770, TRAILR-APC-vio770 (Miltenyi), TLR4-Alexafluor 488 (ebioscience), and IL1RL1-APC (R&D systems). Data were analysed with Flowjo v10.5 (FlowJo, LLC), using the following gating strategy. Doublets were excluded on a forward scatter height / area (FSC-H / FSC-A) dot plot. For the lymphocyte panel (S1 Fig), lymphocytes were first gated on a side scatter area (SSC-A) / FSC-A plot. Next, CD3^+^ and CD3^-^ lymphocytes were gated. Within CD3^+^ lymphocytes, CD4^+^ or CD8^hi^ T cells were selected and CD45RO^+^ memory T cells identified. In parallel, CD3^-^CD8^lo^ lymphocytes were gated as a proxy subset of NK cells, as >99% reportedly express CD56 and CD16 [32]. Finally, the expression of PD-1, KLRG1 and IL-27R was measured in these subpopulations. For the monocyte panel (S2 Fig), monocytes were first gated on a SSC-A / FSC-A plot. CD14^+^ monocytes were gated to determine the expression of TLR2, TLR4, IL1RL1 and TRAILR.

### Ethical considerations

The study was approved by: the ethical review board of Makerere University (IRB-Makerere-05_2007), the institutional review board of the Institute of Tropical Medicine of Antwerp (IRB_ITM_07 25 5 585) and the Ethics Committees of the Faculties of Medicine of the University of Antwerp (CME_UZA_7/29/157). Written informed consent was obtained from all study participants.

### Statistical analysis

Statistics were performed using SPSS software (version 17.0) and GraphPad Prism (version 7) with significance level set at p < 0.05. Differences between patient groups were analysed using One-way ANOVA (for normally distributed data) or Kruskal-Wallis tests (for not normally distributed data). Changes over time were analysed for each group using Friedman tests (n = 8 in each group due to the need for complete follow up samples). Multiple comparison post-hoc tests (Tukey’s and Dunn’s test for normally and not normally distributed data, respectively) and multiplicity adjusted p-values were used to indicate differences between specific groups and time points.

## Results

### Study population

Nested within a prospective cohort study for TB-IRIS at Mulago Hospital in Kampala, Uganda, a total of 13 TB-IRIS patients with PBMCs available prior to ART (n = 13), after 3 months (n = 10) and 9 months (n = 8) of ART were selected and compared to equal numbers of HIV^+^TB^+^ and HIV^+^TB^-^ controls. The median (interquartile range (IQR)) number of days between starting ART and TB-IRIS diagnosis was 14 (13–19) days. No differences in pre-ART clinical characteristics could be observed between any of these patient groups, except for HIV^+^TB^-^ controls who showed lower CRP levels compared to TB-IRIS patients (Table 1). To monitor successful ART, additional CD4 counts were acquired after 6 months. All 3 groups showed a significant increase in CD4 counts by this time (p ≤ 0.007). In addition, 9 HIV^-^TB^-^ controls were selected who did not differ in sex (55% male) or age [median (IQR) age = 36 (31–38)] from any of the HIV-infected groups, and had lower CRP levels [median (IQR) CRP = 0.51 (0.37–0.97)) mg/ml] compared to TB-IRIS patients and HIV^+^TB^+^ controls (p ≤ 0.033).

**Table 1:** Clinical characteristics of the study population.

	TB-IRIS_(n = 13)_	HIV^+^TB^+^_(n = 13)_	HIV^+^TB^-^_(n = 13)_	p
**Pre-ART Characteristics**				
Male (n) (%)	9 (69)	9 (69)	9 (69)	1.000^c^
Age (Years)	38 (30–40)	36 (31–39)	36 (30–41)	0.886
CRP (mg/L)	13.1 (7.5–29.4)	2.75 (0.97–33.47)	2.38 (0.71–3.7)^b^	**≤ 0.001**
Treatment interval^a^	38 (25–71)	58 (37–73)	-	0.411^d^
# days since start TB-treatment	15 (9–28)	9 (7–35)	-	0.538^d^
Extrapulm. TB (%)	6 (46%)	4 (31%)	-	0.420^c^
**# CD4 (cells/μl)**				
Prior to ART	71 (19–120)	80 (35–118)	52 (17–115)	0.785
6 months of ART	204 (147–280)	207 (127–398)	187 (120–307)	0.846

Data are represented as median and interquartile range unless stated otherwise. Kruskal-Wallis tests were used to calculate overall significant differences between groups, unless stated otherwise.

^a^ # days between initiation of TB-treatment and ART.

^b^ Significant difference observed between TB-IRIS and HIV^+^TB^-^ patients according to Dunn’s post-hoc test (p = 0.045).

^c^ Pearson Chi-square test.

^d^ Mann-Whitney U test. The level of significance was set to P > 0.05 for all tests. CRP = C-reactive protein, Extrapulm. TB = extrapulmonary Tuberculosis.

### Expression of KLRG1, PD-1 and IL-27R on memory CD4^+^ T lymphocytes

**Fig 1:**
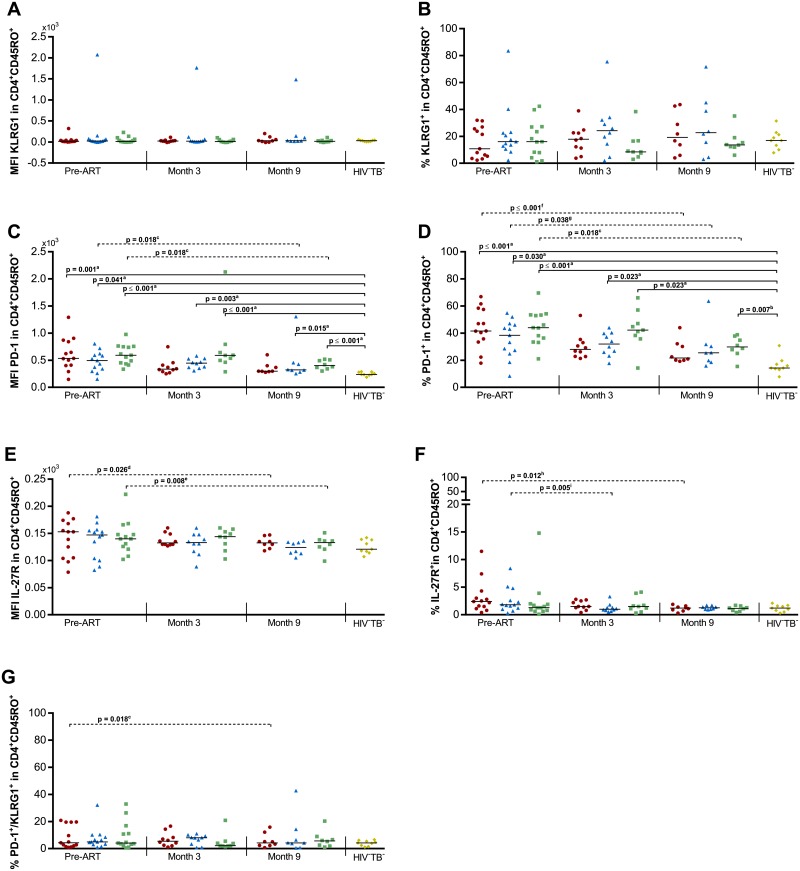
Exhaustion markers on CD4^+^ T cells in TB-IRIS patients and controls. Graphs respectively show the median expression levels (measured by MFI) and the percentage of cells expressing (A-B) KLRG1, (C-D) PD-1, and (E-F) IL-27R within CD45RO^+^CD4^+^ T lymphocytes. In addition percentages are shown of (G) PD-1^+^KLRG1^+^ double positive cells within the CD45RO^+^CD4^+^ T lymphocytes before and during ART in TB-IRIS patients (red circles), HIV^+^TB^+^ (blue triangles), HIV^+^TB^-^ (green squares), and HIV^-^TB^-^ (yellow diamonds). Significant overall differences between groups were calculated using Kruskal-Wallis tests, with Dunn’s post-hoc test shown as capped lines to highlight specific differences between individual groups. Significant variation over time was calculated for each group using Friedman tests, with Dunn’s post-hoc test shown as horizontal dotted lines to highlight specific differences between individual time points. Analysis were performed between all groups at each time point, and between every time points for each group with the level of significance was set to P < 0.05. Non-significant p-values have been omitted from the graphs. Overall Kruskal-Wallis p-values were ^a^p ≤ 0.001, and ^b^p = 0.007 for each set of comparisons. Overall Friedman p-values were ^c^p = 0.018, ^d^p = 0.019, ^e^p = 0.005, ^f^p ≤ 0.001, ^g^p = 0.047, ^h^p = 0.010, and ^i^p = 0.003.

Since CD4^+^ T cell exhaustion is a hallmark of long-term HIV infection, we assessed if the expression patterns of PD-1, KLRG1 and IL-27R on CD45RO^+^CD4^+^ T cells before and during ART differed in HIV-TB patients who did and did not develop TB-IRIS (Fig 1 and S3 Fig). A significant overall difference could be observed between groups in the MFI of PD-1 on CD45RO^+^CD4^+^ T cells prior to ART (p ≤ 0.001), and after 3 months (p ≤ 0.001) and 9 months of ART (p ≤ 0.001). Subsequent post-hoc analysis attributed these differences to significantly lower MFI of PD-1 in HIV^-^TB^-^ controls compared to all groups, except for TB-IRIS at month 3 and month 9 (p ≤ 0.041, Fig 1C). This pattern was mirrored in the percentage of PD-1^+^ cells within CD45RO^+^CD4^+^ T cells (p≤ 0.030, Fig 1D). However, no significant differences could be observed between TB-IRIS patients and HIV^+^TB^+^ controls at any time point. TB-IRIS patients and controls experienced a significant decrease over time of the %PD-1^+^ cells (Friedman test p ≤ 0.047 with Dunn’s post-hoc test p ≤ 0.038, Fig 1D). Moreover, TB-IRIS patients and controls experienced a significant decrease over time in the median fluorescence intensity (MFI) of IL-27R and %IL-27R^+^ cells within CD45RO^+^CD4^+^ T cells (Friedman test p ≤ 0.019 with Dunn’s post-hoc test p ≤ 0.026, Fig 1E and 1F), and only TB-IRIS patients experienced decreasing percentages of PD-1^+^KLRG1^+^ cells within the CD45RO^+^CD4^+^ T cells (Friedman test p = 0.018 with Dunn’s post-hoc test p = 0.018, Fig 1G).

### Expression of KLRG1, PD-1 and IL-27R on memory CD8^hi^ T lymphocytes

**Fig 2:**
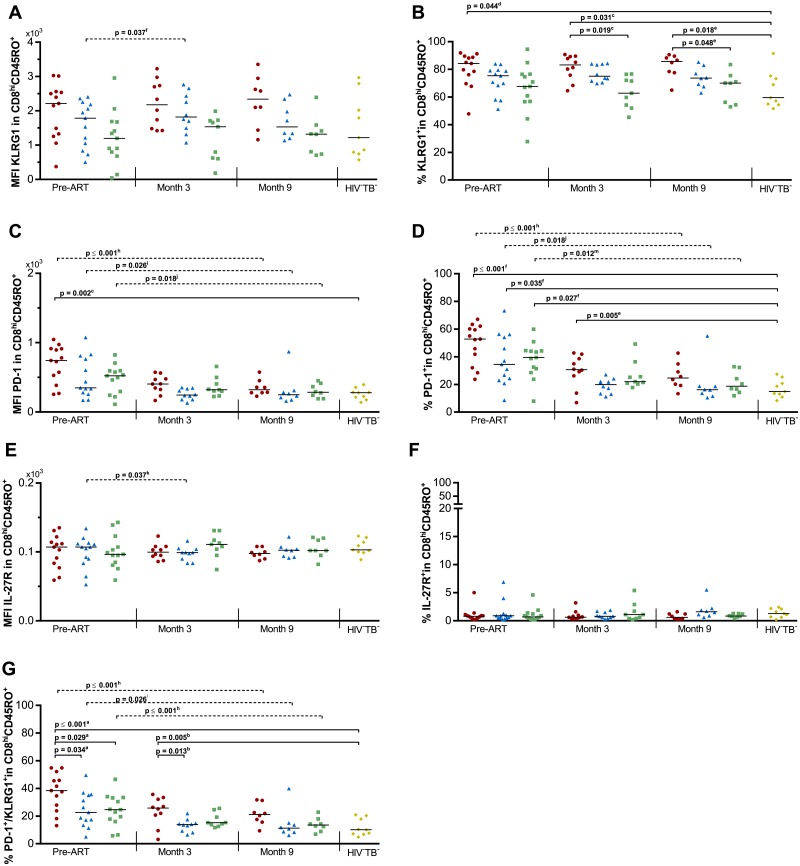
Exhaustion markers on CD8^hi^ T cells in TB-IRIS patients and controls. Graphs respectively show the median expression levels (measured by MFI) and the percentage of cells expressing (A-B) KLRG1, (C-D) PD-1, and (E-F) IL-27R within CD45RO^+^CD8^hi^ T lymphocytes. In addition percentages are shown of (G) PD-1^+^KLRG1^+^ double positive cells within the CD45RO^+^CD8^hi^ T lymphocytes before and during ART in TB-IRIS patients (red circles), HIV^+^TB^+^ (blue triangles), HIV^+^TB^-^ (green squares), and HIV^-^TB^-^ (yellow diamonds). Significant overall differences between groups were calculated using Kruskal-Wallis or One-way ANOVA tests, with Dunn’s or Tukey’s post-hoc test shown as capped lines to highlight specific differences between individual groups. Significant variation over time was calculated for each group using Friedman tests, with Dunn’s post-hoc test shown as horizontal dotted lines to highlight specific differences between individual time points. Analysis were performed between all groups at each time point, and between every time point for each group with the level of significance was set to P < 0.05. Non-significant p-values have been omitted from the graphs. Overall one-way ANOVA p-values were ^a^p ≤ 0.001, and ^b^p = 0.003. Overall Kruskal-Wallis p-values were ^c^p = 0.005, ^d^p = 0.027, ^e^p = 0.014, and ^f^p = 0.003 for each set of comparisons. Overall Friedman p-values were ^g^p = 0.047, ^h^p ≤ 0.001, ^i^p = 0.019, ^j^p = 0.018, ^k^p = 0.032, ^l^p = 0.023, and ^m^p = 0.010.

We next wondered if the expression of PD-1, KLRG1 and IL-27R on CD45RO^+^CD8^hi^ T cells could be associated with TB-IRIS (Fig 2 and S4 Fig). No overall differences could be observed between groups in the expression levels (MFI) of KLRG1 or IL-27R alone. Nonetheless, a significant overall variation in the %KLRG1^+^ cells could be observed at all time points (p ≤ 0.027, Fig 2B). Dunn’s post-hoc analysis attributed this difference to higher percentages in TB-IRIS patients compared to HIV^+^TB^-^ (p ≤ 0.048) and HIV^-^TB^-^ controls (p ≤ 0.044). A significant overall variation in the MFI of PD-1 and the %PD-1^+^ cells could be observed pre-ART (p ≤ 0.014, Fig 2C and 2D). However, Dunn’s post-hoc analysis attributed this difference to lower PD-1 expression in HIV^-^TB^-^ controls compared to other groups (p ≤ 0.035, Fig 2C and 2D). We observed significant overall differences in the percentage of PD-1^+^KLRG1^+^ CD45RO^+^CD8^hi^ T cells prior to ART (p ≤ 0.001, Fig 2G). Tukey’s post-hoc test subsequently showed higher pre-ART percentages in TB-IRIS patients compared to HIV^+^TB^+^ (p = 0.034), HIV^+^TB^-^ (p = 0.029), and HIV^-^TB^-^ controls (p ≤ 0.001). This pattern continued at month 3 (p = 0.003), with higher percentages in TB-IRIS patients compared to HIV^+^TB^+^ (p = 0.013), and HIV^-^TB^-^ controls (p = 0.005). Time analysis showed a significant decrease over time in all HIV-infected groups for PD-1 MFI and the percentage of PD-1^+^ and PD-1^+^KLRG1^+^ cells within the CD45RO^+^CD8^hi^ T cells (Friedman test p ≤ 0.047, with Dunn’s post-hoc test p ≤ 0.026).

### Expression of KLRG1, PD-1 and IL-27R on CD3^-^CD8^lo^ lymphocytes

**Fig 3:**
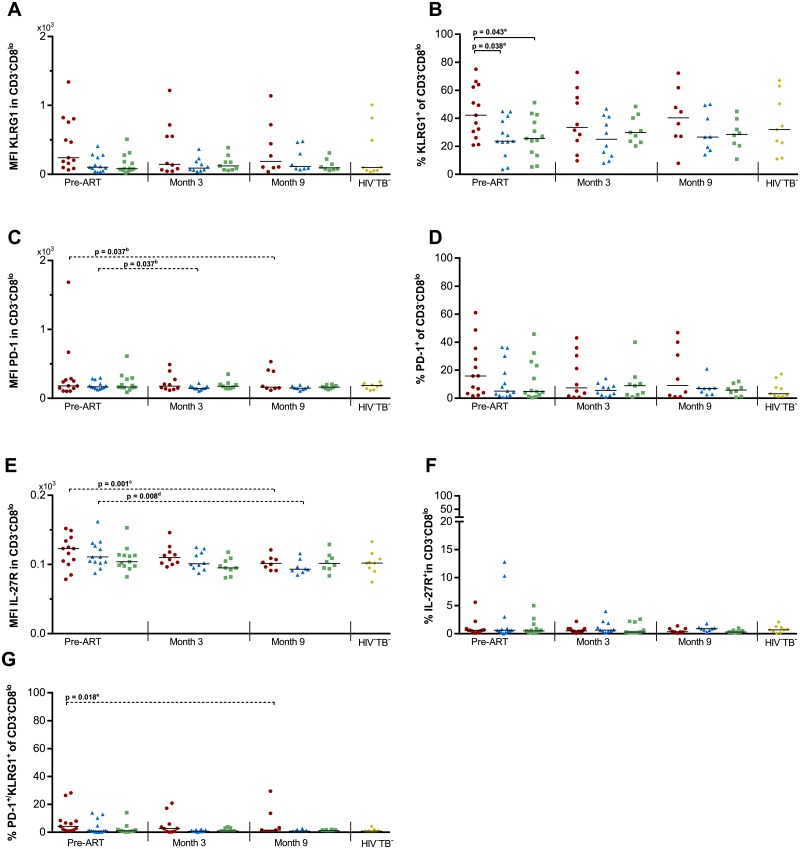
Exhaustion markers on CD3^-^CD8^lo^ lymphocytes in TB-IRIS patients and controls. Graphs respectively show the median expression levels (measured by MFI) and the percentage of cells expressing (A-B) KLRG1, (C-D) PD-1, and (E-F) IL-27R within CD3^-^CD8^lo^ T lymphocytes. In addition percentages are shown of (G) PD-1^+^KLRG1^+^ double positive cells within the CD3^-^CD8^lo^ lymphocytes before and during ART in TB-IRIS patients (red circles), HIV^+^TB^+^ (blue triangles), HIV^+^TB^-^ (green squares), and HIV^-^TB^-^ (yellow diamonds). Significant overall differences between groups were calculated using Kruskal-Wallis or One-way ANOVA tests, with Dunn’s or Tukey’s post-hoc test shown as capped lines to highlight specific differences between individual groups. Significant variation over time was calculated for each group using Friedman tests, with Dunn’s post-hoc test shown as horizontal dotted lines to highlight specific differences between individual time points. Analysis were performed between all groups at each time point, and between every time points for each group with the level of significance was set to P < 0.05. Non-significant p-values have been omitted from the graphs. ^a^One-way ANOVA p = 0.025. Overall Friedman p-values were ^b^p = 0.030, ^c^p ≤ 0.001, ^d^p = 0.010, and ^e^p = 0.018.

Next to CD8^hi^ and CD4^+^ T cells, NK cells have been reported to show signs of exhaustion during HIV [28]. Although no direct NK cell markers were included in our experiments, over 99% of CD3^-^CD8^lo^ lymphocytes have previously been reported to consist of NK cells [32]. We thus further explored the expression of exhaustion markers on these cells, as an approximate subset of NK cells (Fig 3 and S5 Fig). No overall differences could be observed between groups in the MFI of KLRG1, PD-1 or IL-27R. In addition, the percentage of PD-1^+^KLRG1^+^ cells was only modestly higher in TB-IRIS prior to ART, but did not reach significance (overall one-way ANOVA p = 0.057). However, a significant overall difference could be observed prior to ART in the percentage of KLRG1^+^ cells (p = 0.025, Fig 3B), but not PD-1^+^ or IL-27R^+^ cells. Tukey’s post-hoc test next showed significantly higher percentages in TB-IRIS patients comparted to HIV^+^TB^+^ (p = 0.038), and HIV^+^TB^-^ controls (p = 0.043). Time analysis showed a significant decrease over time in TB-IRIS patients and HIV^+^TB^+^ controls for the MFI of PD-1 and IL-27R, and the percentage of PD-1^+^KLRG1^+^ cells within the CD3^-^CD8^lo^ lymphocytes (Friedman test p ≤ 0.030, with Dunn’s post-hoc test p ≤ 0.037).

### Expression of TLR2, TLR4, IL1RL1 and TRAILR on monocytes

**Fig 4:**
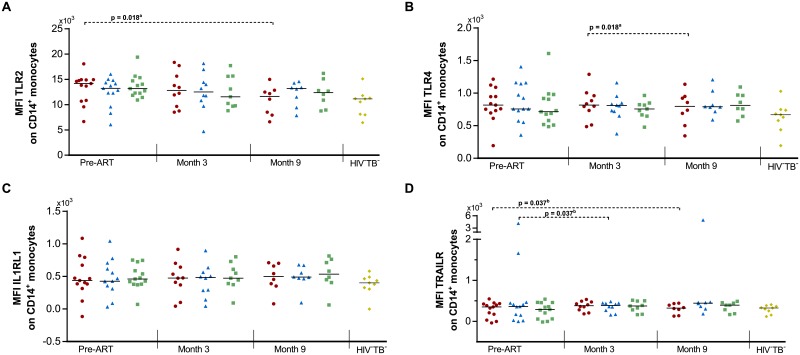
Expression of TLR2, TLR4, IL1RL1, and TRAILR on monocytes in TB-IRIS patients and controls. Graphs show the expression of (A) TLR2, (B) TLR4, (C) IL1RL1, and (D) TRAILR on monocytes before and during ART in TB-IRIS patients (red circles), HIV^+^TB^+^ (blue triangles), HIV^+^TB^-^ (green squares), and HIV^-^TB^-^ (yellow diamonds). Significant overall differences between groups were calculated using Kruskal-Wallis tests, with Dunn’s post-hoc test shown as capped lines to highlight specific differences between individual groups. Significant variation over time was calculated for each group using Friedman tests, with Dunn’s post-hoc test shown as horizontal dotted lines to highlight specific differences between individual time points. Analysis were performed between all groups at each time point, and between every time points for each group with the level of significance was set to P < 0.05. Non-significant p-values have been omitted from the graphs. ^a^One-way ANOVA p = 0.047. Overall Friedman p-values were ^a^p = 0.018, and ^b^p = 0.030.

To further evaluate the potential role of monocytes in TB-IRIS, we next examined the expression of TLR2, TLR4, IL1RL1 and TRAILR on CD14^+^ monocytes (Fig 4 and S6 Fig). However, no differences could be observed between TB-IRIS patients and HIV^+^TB^+^ or HIV^+^TB^-^ controls. Time analysis showed a significant decrease over time in TB-IRIS patients in the MFI of TLR2 and TLR4 (Friedman test p = 0.018 & Dunn’s post-hoc test p = 0.018 for both markers) and in the MFI of TRAILR for TB-IRIS patients and HIV^+^TB^+^ controls (Friedman test p = 0.030 & Dunn’s post-hoc test p = 0.037 for each group).

## Discussion

Decades of research into TB-IRIS have highlighted potential roles of the innate and the adaptive immune system in the inflammatory cascade that characterizes the disease [11,12,16,17]. Nonetheless, the pre-ART mechanisms that predispose the immune system to hyper-react upon ART initiation remain unclear. Despite being a well-documented risk factor, low pre-ART CD4 counts alone cannot account for all TB-IRIS cases. Since up to 25% of HIV-TB patients with severe immunosuppression develop TB-IRIS [2], other antecedent factors have to be at play in order for this complication to occur. We hypothesised that higher levels of T cell exhaustion prior to ART would predispose HIV-TB patients to develop TB-IRIS. Here, we describe higher levels of exhaustion in CD8^hi^ T cells and a subpopulation of NK cells, but not CD4^+^ T cells, which precede and persist after TB-IRIS. In severely immunocompromised individuals, the functionality of these cells may thus be an additional determining factor in TB-IRIS development.

Given the obvious link between low CD4 counts prior to ART and TB-IRIS, we first assessed the exhausted phenotype of CD4^+^ T cells in TB-IRIS patients. Contrary to our hypothesis but in line with previous studies [23,24], we could not observe any significant differences in CD4^+^ T cell phenotypes that singled out TB-IRIS patients from the HIV^+^ control groups. It is therefore unlikely that CD4^+^ T cell exhaustion specifically contributed to TB-IRIS development in our cohort. Corresponding to our hypothesis, however, the frequency of memory CD8^hi^ T cells which co-expressed KLRG1 and PD-1 was much higher in TB-IRIS patients before and after starting ART. This finding corresponds to a previous report of higher frequencies of PD-1^+^CD8^+^ T cells in non-pathogen-specific IRIS, although the same was observed for CD4^+^ T cells [33]. Importantly, the exhaustion levels in TB-IRIS patients did not normalize to the level of HIV-negative controls by 3 months of ART, and trended to be remain higher after 9 months, suggesting a more long-lasting effect on the immune system. High levels of immune exhaustion have typically been observed after prolonged periods of antigenic stimulation, in particular with infections such as TB [27] and HIV [34]. It is therefore plausible that a high antigen load at an earlier stage may have driven CD8^+^ specific exhaustion, perhaps even leading to an anergic state of these cells before ART was administered. Of note, TB-IRIS patients in our study showed modestly higher CRP levels prior to ART, and a slightly shorter treatment interval, which could indicate higher antigen levels. However, these differences did not reach statistical significance compared to HIV^+^TB^+^ controls.

Interestingly, our findings on CD8^hi^ T cells were mirrored by innate lymphocytes as well. Indeed, we observed higher pre-ART frequencies of KLRG1^+^CD8^lo^ lymphocytes that did not express CD3. CD3^-^CD8^lo^ lymphocytes have previously been reported to mainly consist of NK cells [32]. In addition to CD8^hi^ T cells, TB-IRIS patients in our cohort thus also experienced exhaustion in a subset of the NK cell compartment prior to ART. Both CD8^+^ T cells and NK cells drive immune responses against intracellular pathogens such as HIV and common co-infections such as TB and cytomegalovirus (CMV), either directly through cytotoxicity or indirectly by interferon-gamma (IFN-γ) mediated stimulation of monocytes/macrophages. A decrease in their function as a consequence of exhaustion could thus hypothetically result in the accumulation of intracellular antigenic signals prior to ART. Although these results advocate for an additional role of the innate immune system in TB-IRIS, we could not observe differential expression of TLR2, TLR4, IL1RL1 or TRAILR on monocytes in TB-IRIS patients. While our results thus cannot confirm an active participation of monocytes in TB-IRIS development prior to ART, they do not exclude a role for monocytes during TB-IRIS, as suggested by previous studies [35,36].

Taken together, the immune-exhaustion observed here could be a consequence of increased pre-ART antigen loads, which have been associated with TB-IRIS before [30]. However, as we did not observe exhaustion on CD4^+^ T cells, our results suggest a specific role for lymphocytes that express CD8 in the early predisposition to TB-IRIS. One of the more attractive theories to date states that a lack of CD4^+^ T cell help in severely immunocompromised individuals could lead to priming of the innate immune system by an increased antigen load. Initiation of ART and the subsequent rise in IFN-γ could thus trigger an excessive response to these accumulated antigenic signals [5]. Nonetheless, even with severe immunosuppression, not all patients develop TB-IRIS. Building on this theory, we now hypothesise that TB-IRIS might find its origins in a form of “immune paralysis” prior to ART. Infections such as HIV, TB and CMV are highly co-endemic, result in long-term infection and are known to cause CD8^+^ T cell exhaustion. Given time, continuous stimulation by any of these pathogens could push CD8^+^ T cells and NK cells past normal activation, into a stage of dysfunctional exhaustion. When CD4 counts reach dangerous levels, the resulting lack of cytotoxicity and/or IFN-γ signalling would prime innate cells with ever increasing antigenic signals, to be unleashed upon ART. HIV-TB patients who do not experience TB-IRIS may not yet have reached an exaggerated level of exhaustion due to less pronounced or shorter exposure to these pathogens.

Due to the retrospective nature of our study, one limitation was the availability of frozen PBMCs. Moreover, the unpredictability of TB-IRIS resulted in a limited number of samples collected during the TB-IRIS event of every patient even further. As such, frozen PBMCs taken at follow-up time points where not available for each single patient included in this study. We thus cannot provide insight in the role of immune exhaustion during the ongoing TB-IRIS inflammation. Nonetheless, 8 out of the 13 patients in each group did have samples available at time points that span a timeframe both before and after TB-IRIS development. This allows for a broad overview of the process leading up to IRIS, as well as the recovery after. While the resulting study population may have been underpowered to detect certain effects (e.g. TLR2 expression on monocytes), the effects observed on immune-exhaustion are thus likely stronger than observed here.

In conclusion, we report high frequencies of exhausted (KLRG1^+^PD-1^+^) CD8^hi^ T cells and (KLRG1^+^) CD3^-^CD8^lo^ cells which precede TB-IRIS and, in the case of CD8^hi^ T cells, persist during ART. In contrast, we observed no evidence of altered CD4^+^ T cell or monocyte phenotypes prior to ART. Our findings thus suggest that higher levels of exhaustion in cells specialised in intracellular immunity predispose HIV-TB patients to TB-IRIS. Although a role for the innate immune system in TB-IRIS should not be excluded, the potential causal link with cytotoxic cells prior to ART merits further investigation. Resolving the immune responses leading to TB-IRIS pathogenesis could guide a targeted approach to accurately predict TB-IRIS in patients at risk.

## Supporting information

S1 Fig Gating strategy for the analysis of exhaustion markers on lymphocytes (panel 1). (TIFF)

S2 Fig Gating strategy for the analysis of markers on monocytes (panel 2). (TIFF)

S3 Fig Representative plots for the expression of exhaustion markers on CD4^+^ T cells. (TIFF)

S4 Fig Representative plots for the expression of exhaustion markers on CD8^+^ T cells. (TIFF)

S5 Fig Representative plots for the expression of exhaustion markers on CD3^-^CD8^lo^ cells. (TIFF)

S6 Fig Representative histograms for the expression of markers on CD14^hi^ monocytes. (TIFF)
