# Supplementary figures and images for: Increased KLRG1 and PD-1 expression on CD8 T lymphocytes in TB-IRIS
Source: PLoS One. 2019 Apr 25;14(4):e0215991. doi: 10.1371/journal.pone.0215991 (PMC6483230; doi:10.1371/journal.pone.0215991)

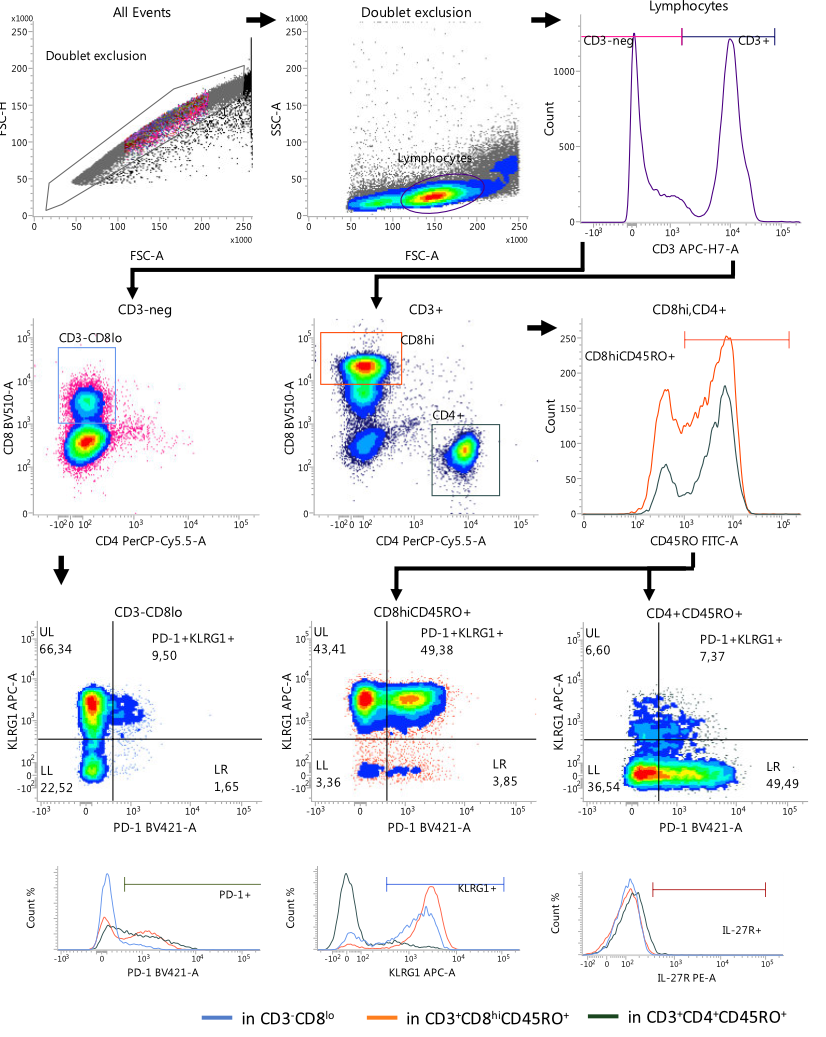

Supplement: S1 Fig — (TIFF) [file pone.0215991.s001.tiff]

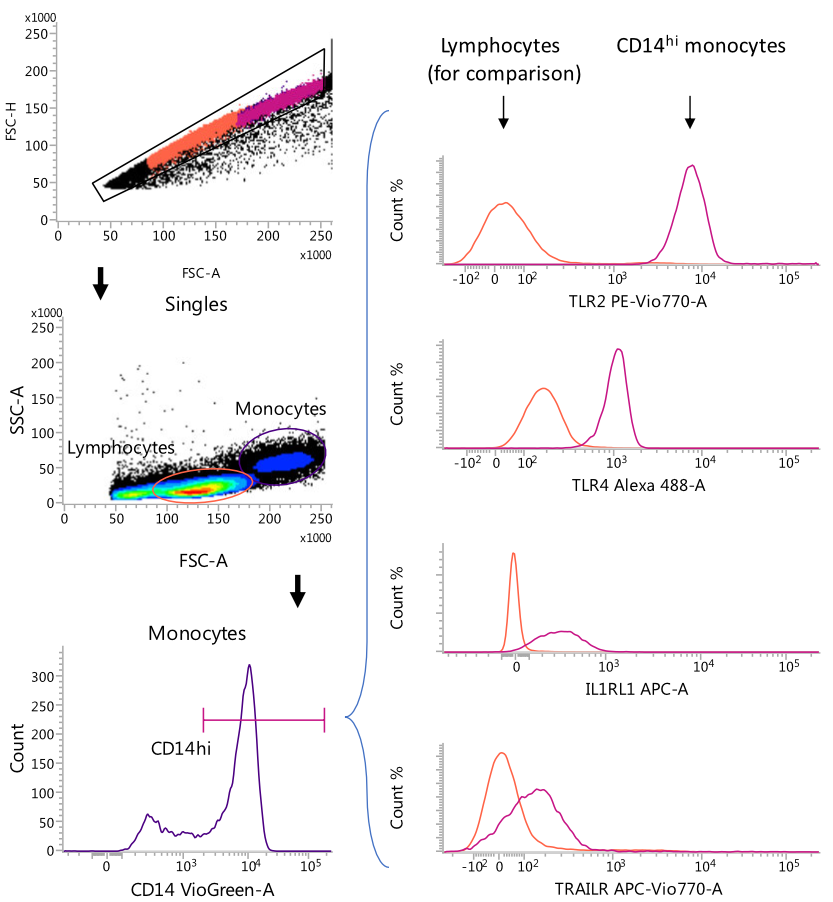

Supplement: S2 Fig — (TIFF) [file pone.0215991.s002.tiff]

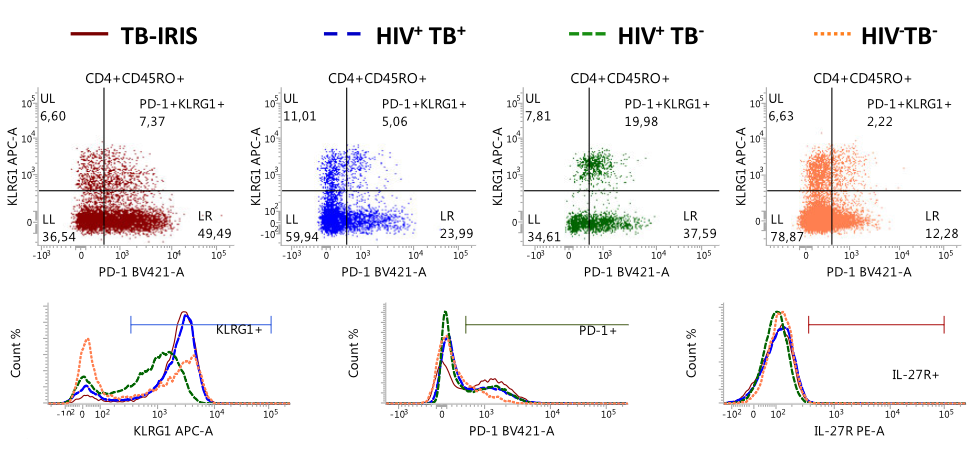

Supplement: S3 Fig — (TIFF) [file pone.0215991.s003.tiff]

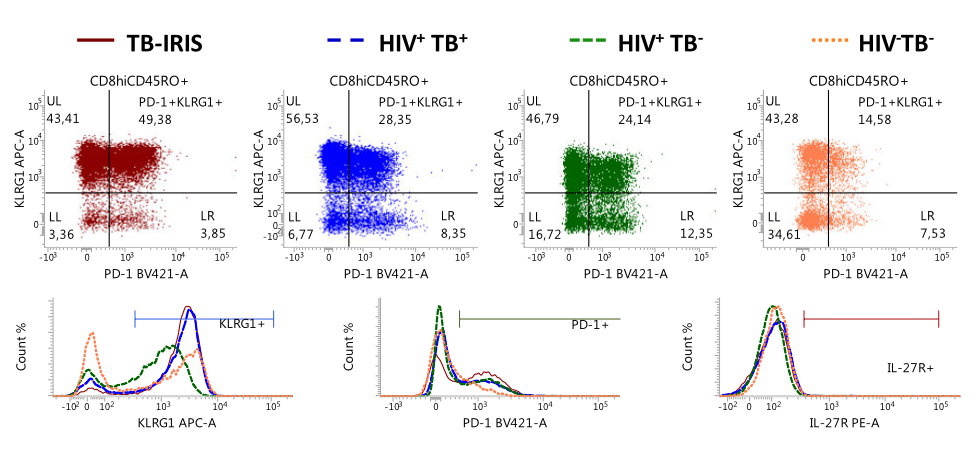

Supplement: S4 Fig — (TIFF) [file pone.0215991.s004.tiff]

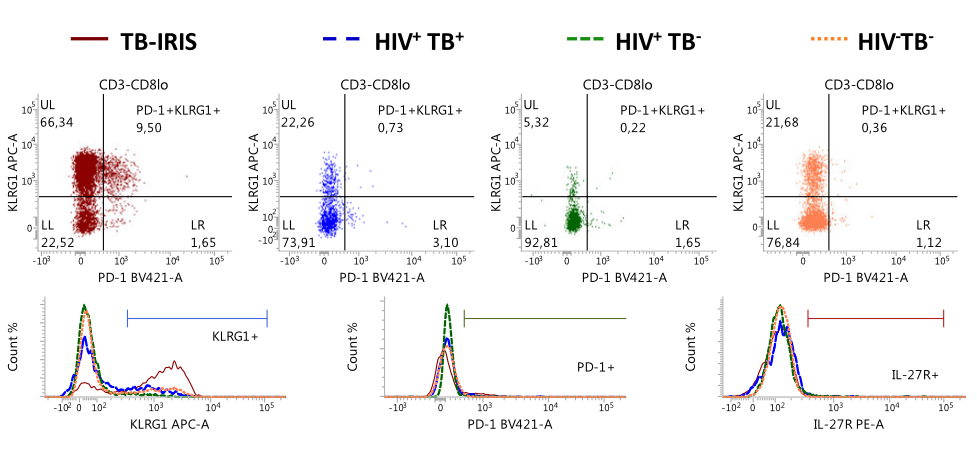

Supplement: S5 Fig — (TIFF) [file pone.0215991.s005.tiff]

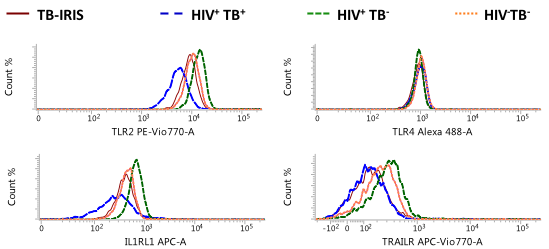

Supplement: S6 Fig — (TIFF) [file pone.0215991.s006.tiff]
